# Effectiveness of proprioceptive training and conventional physical therapy in treating adhesive capsulitis

**DOI:** 10.12669/pjms.37.4.3874

**Published:** 2021

**Authors:** Rida Shabbir, Aatik Arsh, Haider Darain, Sadaf Aziz

**Affiliations:** 1 Rida Shabbir, DPT, MSPT. Lecturer, Institute of Physical Medicine and Rehabilitation, Khyber Medical University Peshawar, Pakistan; 2 Aatik Arsh, DPT, MSPT. Lecturer, Institute of Physical Medicine and Rehabilitation, Khyber Medical University Peshawar, Pakistan; 3 Haider Darain, BSPT, MSPT, PhD. Professor, Institute of Physical Medicine and Rehabilitation, Khyber Medical University Peshawar, Pakistan; 4 Sadaf Aziz, DPT, MSPT. Assistant Professor, Rehman College of Rehabilitation Sciences, Peshawar, Pakistan

**Keywords:** Frozen Shoulder, Pain, Physical Therapy, Proprioception, Rehabilitation

## Abstract

**Objective:**

To determine effectiveness of proprioceptive training and conventional physical therapy in managing patients with adhesive capsulitis.

**Methods:**

A pre-test post-test control group study was conducted at Rehman Medical Institute from June to December 2019. Thirty-eight patients, aged 30 to 60 years, with diagnosis of adhesive capsulitis for more than four months were divided into two groups. Subjects in Group-I (conventional group; n=19) received conventional physical therapy protocols for one month while subjects in Group-II (proprioceptive group; n=19) received proprioceptive training along with conventional physical therapy for one month. Disability of Arm Shoulder & Hand questionnaire, Shoulder Pain & Disability Index and goniometer were used at baseline and post-treatment to assess functional activity level, pain & disability and range of motion respectively. Data was analyzed using SPSS version 20.

**Results:**

The mean age of the participants was 53.13 ± 9.12 years. Baseline characteristics were balanced between the two groups. After the treatment, all measures (functional activity level, pain, disability, ROM) improved in both groups. Post treatment, between group analysis showed that functional activity (DASH) and pain (SPADI pain) significantly (P-value <0.05) improved in proprioceptive group as compared to conventional group. However, there were no significant differences (P-value≥ 0.05) in post treatment SPADI disability, SPADI total and ROM (flexion, abduction, external rotation) scores of both groups.

**Conclusion:**

Proprioceptive exercises along with conventional physical therapy are more effective in managing pain and improving functional activities in adhesive capsulitis patients as compared to conventional physical therapy alone.

## INTRODUCTION

Adhesive capsulitis is a common musculoskeletal disorder characterized by reduced passive and active shoulder range of motion (ROM).^1^ It results in progressive pain with loss of mobility of the involved extremity and inability to sleep on effected side Adhesive capsulitis is classified as primary adhesive capsulitis^2^, with no histological and radiographic findings other than osteopenia and calcific tendonitis, and secondary adhesive capsulitis, with known cause of stiffness and decreased ROM.^3^ Adhesive capsulitis can lead to contractures of glenohumeral joint capsule causing decreased capsular extensibility, adherent axillary recess, proprioceptive loss and decreased extensibility of bicep tendon.^4^,^5^

A number of physical therapy interventions can be used for the management of adhesive capsulitis. Maitland mobilizations, short wave diathermy, stretching exercises, hot pack, soft tissue mobilization, and therapeutic exercises are some of the commonly used interventions for treating adhesive capsulitis patients.^6^ In recent years proprioceptive exercises received great attention from clinicians in managing shoulder joint conditions.^7^ Glenohumeral joint, along with its muscles, ligaments and joint capsule, are richly supplied by mechano-receptors that detects kinesthesia and joint position. Capsular lesions of the shoulder joint decreases sensitivity of the receptors which can lead to loss of proprioceptive and neurological feedback responses.^8^

Proprioceptive training using plyometrics has been studied in acute traumatic joint pathologies, degenerative joint pathologies and in athletic population which leads to improved proprioceptive feedback and kinesthesia. It is due to peripheral adaptation that occurs due to stimulation of articular mechanoreceptors repeatedly.^9^ Despite the fact that many studies has evaluated effects of proprioceptive exercises in the management of different shoulder pathologies, however, literature is scarce regarding effectiveness of proprioceptive exercises in treating adhesive capsulitis.^7^,^10^,^11^ Therefore, current study was designed to determine the effectiveness of proprioceptive training and conventional physical therapy in managing patients with adhesive capsulitis.

## METHODS

A pre-test post-test control group study was conducted at Rehman Medical Institute (RMI) Peshawar from June to December 2019. Ethical approval was obtained from KMU Ethics Board (DIR/KMU-EB/AR/000388). Male and female patients, aged 30 to 60 years, with diagnosis of adhesive capsulitis for more than four months were included through consecutive sampling. Patients with bilateral adhesive capsulitis, unstable fractures, rheumatoid arthritis and those with severe joint pain unrelieved by rest were excluded from the study. Diagnosis of adhesive capsulitis was confirmed on the basis of thorough physical examination. Painful and limited active and passive glenohumeral ROM >25% in capsular pattern (external rotation then abduction and then flexion) was considered diagnostic criteria for adhesive capsulitis. Physical tests such as Hawkins-Kennedy test, empty can test etc., and radiographs were used to exclude other shoulder conditions.

Informed consent was obtained from subjects who fulfill the eligibility criteria and were willing to participate in the study. Participants were divided into two groups through sealed envelope method. Subjects in Group-I (Conventional group) received conventional physical therapy including stretches for levator scapula, upper trapezius, pectoralis minor and major using holds for 10 seconds and then stretch for 10 seconds, five times per session. Strengthening exercises were given for middle and lower trapezius and rotator cuff by giving manual resistance for 10 seconds for each muscle, 5 x 2 times per session. Shoulder anteroposterior, posterioanterior and inferior glides were given 30 x 3 in grade III and IV. Each session was of 35 to 50 minutes. Besides conventional physical therapy, subjects in Group-II (Proprioceptive group) received proprioceptive training such as plyometrics for shoulder joint including closed kinetic chain exercises for first week where patient were quadruped on the ground with involved extremity fixed to the ground and uninvolved extremity being held in flexion and abduction, 10 seconds each for 5 x 2. Closed kinetic chain exercises on ball were given on second and third week where patient was quadruped on the ground with involved extremity on ball and uninvolved extremity being half in flexion and abduction for 10 seconds each for 5 x 2. In the fourth week, patient received open kinetic chain exercises where they threw the ball against the wall while keeping the arm in internal and external rotation. Each session was of 35 to 50 minutes. Both groups received 16 sessions (4 sessions per week) spread over four weeks.

Disability of Arm Shoulder & Hand (DASH) questionnaire, Shoulder Pain & Disability Index (SPADI) and goniometer were used at baseline and post-treatment (at the end of 4^th^ week of treatment) to assess functional activity level, pain & disability and ROM (flexion, abduction and external rotation), respectively. Data was analyzed using SPSS version 20. Shapiro-Wilk test was used to assess normality of the data. Because data was normally distributed that is why Independent sample t-test was used for to analyze the difference between the two groups. P-value of <0.05 were considered significant.

## RESULTS

A total of 38 subjects (13 (34.2%) male & 25 (65.8%) female) participated in the study. The mean age of the participants was 53.13 ± 9.12. There were 19 participants in each group. Baseline characteristics were balanced between the two groups. There were no significant differences in pretreatment DASH, SPADI (pain, disability, total score) and ROM (flexion, abduction, external rotation) scores of conventional and proprioceptive groups (Table-I).

**Table-I:** Pre-treatment Scores of Conventional and Proprioceptive groups.

Variable	Conventional grou Mean ± S.D	Proprioceptive group Mean ± S.D	P-Value
Pre DASH	63.79 ± 12.29	56.88 ± 16.90	0.158
Pre SPADI Pain	70.42 ± 7.88	67.26 ± 15.45	0.433
Pre SPADI Disability	67.37 ± 10.67	57.50 ± 16.76	0.098
Pre SPADI Total	68.54 ± 9.64	61.30 ± 15.18	0.088
Pre Flexion ROM	92.53 ± 13.24	85.52 ± 26.48	0.310
Pre Abduction ROM	68.89 ± 9.65	66.42 ± 29.14	0.727
Pre External Rotation ROM	36.68 ± 10.88	34.32 ± 10.70	0.503

DASH: Disability of Arm Shoulder & Hand, ROM: Range of Motion, SD: Standard Deviation, SPADI: Shoulder Pain & Disability Index

After the treatment, all measures (functional activity level, pain, disability, ROM) improved in both groups. Post treatment, between group analysis showed that functional activity (DASH) and pain (SPADI pain) significantly (P-value <0.05) improved in proprioceptive group as compared to conventional group. However, there were no significant differences (P-value≥ 0.05) in post treatment SPADI disability, SPADI total and ROM (flexion, abduction, external rotation) scores of both groups (Table-II).

**Table-II:** Post-treatment Scores of Conventional and Proprioceptive groups.

Variables	CONVENTIONAL Mean ± S.D	PROPRIOCEPTIVE Mean ± S.D	P-Value
Post dash	37.65 ± 12.05	27.81 ± 13.68	0.020
Post SPADI Pain	54.42 ± 9.15	39.67 ± 18.78	0.020
Post SPADI Disability	50.00 ± 10.52	30.88 ± 15.11	0.319
Post SPADI Total	51.70 ± 9.80	32.69 ± 14.82	0.273
Post Flexion ROM	126.63 ± 24.34	127.42 ± 37.36	0.055
Post Abduction ROM	107.89 ± 24.46	110.36 ± 32.18	0.953
Post External Rotation ROM	55.53 ± 11.01	58.37 ± 9.63	0.538

## DISCUSSION

Adhesive capsulitis, commonly known as frozen shoulder is a costly and disabling musculoskeletal condition.^12^ Current study was undertaken to evaluate the effectiveness of proprioceptive training and conventional physical therapy in reducing pain & disability and increasing ROM of shoulder joint in adhesive capsulitis patients. The study showed functional activity level, pain, disability and ROM improved in adhesive capsulitis patients who either received conventional physical therapy or proprioceptive training. Nevertheless, functional activity and pain were significantly more improved in patients who received proprioceptive training as compared to patients who receive conventional physical therapy alone.

According to author’s knowledge, current study was one of the preliminary studies which assessed effects of proprioceptive exercises in adhesive capsulitis patients. McMullen et al. studied kinetic chain shoulder rehabilitation and proximal to distal muscular activation pattern using proprioceptive neuromuscular facilitation (PNF) and closed kinetic chain exercises to improve strength and ROM of shoulder musculature.^13^ They speculated that kinetic chain exercises improve strength and ROM. The present study has also used kinetic chain exercises for the subjects in proprioceptive training where functional activities, pain and ROM improved significantly however there was not any difference seen between proprioceptive group and conventional exercises group in terms of ROM. The reason for this difference can be due to the fact that McMullen et al. reported generalized effects of kinetic chain exercises for all shoulder disorders while the present study only focused on the adhesive capsulitis. Balci et al. had studied the effects of scapular PNF and conventional exercises in adhesive capsulitis. They concluded that pain improved significantly in proprioceptive group but there were no significant differences between the two groups. ROM of flexion and abduction did not improve significantly.^5^ These results are consistent with our study where pain improved significantly in proprioceptive group and ROM showed no significant difference. Though open and closed chain exercises were not used by Balci et al. but they worked on proprioception which showed consistent results with the current study. Prasanna et al. had studied the effectiveness of PNF in frozen shoulder and compared it with conventional physical therapy. Their study concluded that scapular PNF techniques along with the shoulder joint stretches and mobilization is effective in the management of the adhesive capsulitis by reducing pain, increasing ROM and functional abilities.^14^ The findings of this study can be compared with the present study as joint mobilization, stretches, and proprioceptive exercises were given to the subjects but the contrasting factor is that instead of open and closed kinetic chain exercises, scapular PNF techniques were given in the study of Prasanna et al. Both the studies reported that proprioceptive exercises reduced pain, increased ROM of abduction and external rotation and functional abilities.

It is pertinent to mention that conventional physical therapy interventions including electrotherapy, manual therapy, exercise therapy is widely described in the literature for the management of adhesive capsulitis. Quite a few studies have reported effectiveness of these physical therapy modalities for treating adhesive capsulitis patients.^15^-^17^ Current study was no different and reported that conventional physical therapy techniques can be used to manage patients with adhesive capsulitis. Physical therapy decreases pain, improves ROM and therefore increases functional abilities. A systematic review conducted by Jain & Sharma reported that joint mobilization and therapeutic exercises given as physical therapy treatment are highly recommended in order to improve range, decrease pain and improve activities of daily living in individuals suffering from adhesive capsulitis.^18^,^19^

### Limitations of the study

Despite the fact that current study was a preliminary study, which evaluated effectiveness of proprioceptive exercises in treating adhesive capsulitis, yet it has some limitations. First of all, current study was conducted in clinical settings, so it was not possible to control confounding variables. Secondly, Blinding was not ensured which can bias the results of current study. Moreover, due to small sample size, result of current study cannot be generalized.

## CONCLUSION

It can be concluded that conventional physical therapy and proprioceptive exercises improve pain, disability, functional activities and ROM in patients with adhesive capsulitis, however, proprioceptive exercises along with conventional physical therapy is more effective in managing pain and improving functional activities as compared to conventional physical therapy alone. Large, multicenter, randomized controlled trials are recommended to truly determine effectiveness of proprioceptive exercises in managing adhesive capsulitis patients.

### Authors` Contribution:

**RS:** Concept and study design, literature search and literature review, acquisition of data, drafting the manuscript.

**AA:** Concept and study design, data analysis and interpretation, drafting the manuscript, final approval of the version to be published. He is responsible for the accuracy or integrity of the work.

**HD:** Literature search and literature review, data analysis and interpretation, critical revision.

**SA:** Acquisition of data, data analysis and interpretation, critical revision.
